# Epidemiology of scarlet fever in Victoria, Australia, 2007–2017

**DOI:** 10.1017/S0950268824001298

**Published:** 2024-10-04

**Authors:** Sachin Phakey, Patricia T. Campbell, Katherine B. Gibney

**Affiliations:** 1 The Royal Melbourne Hospital, Melbourne, VIC, Australia; 2 Department of Infectious Diseases, The University of Melbourne at The Peter Doherty Institute for Infection and Immunity, Melbourne, VIC, Australia

**Keywords:** epidemiology, Group A *Streptococcus*, scarlatina, scarlet fever, *Streptococcus pyogenes*

## Abstract

In the last 10–15 years, there has been a global resurgence of scarlet fever, an infection historically associated with significant morbidity and mortality. It is unknown whether scarlet fever incidence has increased in Australia. We aimed to examine the incidence, predictors and severity of scarlet fever in the state of Victoria, Australia from 2007 to 2017, analyzing scarlet fever emergency department (ED) presentations, hospitalizations and deaths. Of the 1 578 scarlet fever cases during the study period, most occurred in children aged <10 years (1 344, 85%), in males (882, 56%), and during winter and spring months (918, 57%). There were no deaths with scarlet fever, however, 374 cases (24%) were admitted to hospital. The annual incidence of scarlet fever was stable during the study period (mean, 2.5; range, 1.9–3.1 cases per 100 000). Annual incidence was highest in children aged <5 years (19.3 per 100 000), and was 21% higher in males than females, adjusting for age and year (incidence rate ratio, 1.21, 95%CI 1.09–1.34). Whilst scarlet fever ED presentations and hospitalizations were stable in Victoria from 2007 to 2017, the recent identification of a *Streptococcus pyogenes* variant in Australia associated with epidemic scarlet fever overseas highlights the risk of future outbreaks.

## Introduction

Scarlet fever is a toxin-mediated manifestation of *Streptococcus pyogenes* (Group A *Streptococcus*, GAS) infection, responsible for considerable morbidity and mortality in the 19th and early 20th centuries, when its incidence was high [1, 2]. Whilst the incidence of scarlet fever subsequently declined, there has been a global resurgence in the last 10–15 years, particularly in Asia during 2005–2015 [1–4]. It is unclear whether the incidence of scarlet fever is increasing in Australia. To our knowledge, there have only been two studies which describe the epidemiology of scarlet fever in Australia [5, 6], both limited by small sample sizes (*n* < 15), short study periods and non-population-based designs. Further data are required to elucidate the epidemiology of scarlet fever in Australia. Such information would be useful to help guide public health policy and resource allocation, particularly given scarlet fever’s potential morbidity and global resurgence. The aim of this study was to examine the incidence, predictors and severity of scarlet fever in Victoria, Australia’s second most populous state, from 2007 to 2017. The study period coincides with the resurgence of scarlet fever in Asia, and focuses on the period prior to the coronavirus disease 2019 (COVID-19) pandemic. Since recent data from Australia and elsewhere suggests the epidemiology of GAS may have been disrupted during COVID-19 [7, 8], this pre-pandemic analysis provides a baseline, against which future incidence can be compared.

## Methods

We performed a retrospective population-based analysis of routinely collected, administrative health data. We analyzed emergency department (ED) presentations, hospital admissions, and deaths with scarlet fever in Victoria, Australia using three linked datasets: the Victorian Emergency Minimum Dataset; the Victorian Admitted Episodes Dataset; and the National Cause of Death Unit Record File, from 1 January 2007 to 31 December 2017. Scarlet fever cases and deaths were identified by the International Classification of Disease (ICD-10) code, A38. We calculated annual incidence rates of scarlet fever using the Australian Bureau of Statistics’ mid-year estimated resident population data, and incidence rate ratios (IRR) and their 95% confidence intervals (CI) using Poisson regression. Multivariable analysis included sex, age group and year, generating adjusted IRRs (aIRR) and 95% CIs. We formally tested for seasonal variation using Edwards’ test (Supplementary Methods). Ethics approval was obtained from The University of Melbourne Human Research Ethics Committee (reference, 2021-12232-14388-3).

## Results

From 2007 to 2017, there were 1 578 ED presentations and hospital admissions with scarlet fever identified in Victoria, Australia. Of these, 1 344 (85%) occurred in children aged <10 years and 882 (56%) were male (Table 1). Approximately one in four cases (374, 24%) were admitted to hospital, however, few (177, 11%) were admitted for two or more days (Table 1, Supplementary Results). There were no deaths with scarlet fever.

**Table 1.** Scarlet fever emergency department presentations and hospitalisations, Victoria, Australia, 2007–2017, by sex and age group

	No. of cases (%)	Mean annual incidence (per 100 000)	Univariable analysis IRR (95% CI)	Multivariable analysis aIRR (95% CI)
Total	1 578 (100)	2.5	–	–
Sex				
Female	696 (44)	2.2	Reference	Reference
Male	882 (56)	2.9	1.29 (1.17–1.43)	1.21 (1.09–1.34)
Age group (years)				
0–4	775 (49)	19.3	Reference	Reference
5–9	569 (36)	14.8	0.77 (0.69–0.86)	0.77 (0.69–0.86)
10–19	139 (9)	1.8	0.09 (0.08, 0.11)	0.09 (0.08–0.11)
≥20	95 (6)	0.4	0.01 (0.008–0.013)	0.01 (0.008–0.013)
Year	–	–	1.00 (0.98–1.01)	0.99 (0.98–1.01)

aIRR, adjusted incidence rate ratio (adjusting for sex, age group and year); CI, confidence interval; IRR, incidence rate ratio.

**Figure 1.**
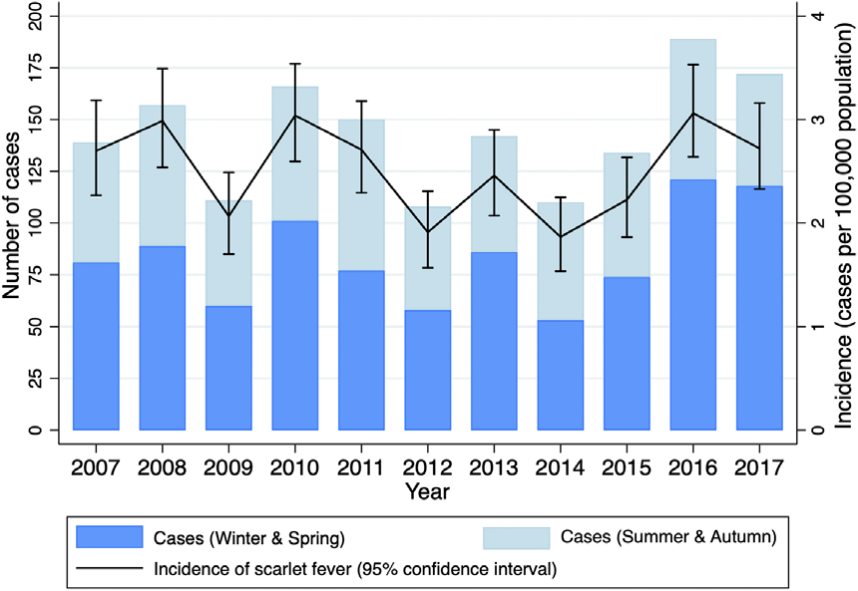
Annual scarlet fever hospital presentations (emergency department and hospitalization admissions), Victoria, Australia, 2007–2017.

The annual incidence of scarlet fever was generally stable from 2007 to 2017 (Figure 1), ranging from 1.9 cases per 100 000 in 2012 and 2014 to 3.1 cases per 100 000 in 2016 (mean, 2.5 per 100 000). There was no significant trend in annual incidence across the study period, adjusting for sex and age group (aIRR, 0.99, 95%CI 0.98–1.01) (Table 1). Most cases (918, 57%) occurred during the winter and spring months, June–November (Figure 1), with Edwards’ test suggesting a seasonal tendency, albeit the goodness-of-fit was equivocal (Χ^2^ = 309.8, *p* < 0.001).

The annual incidence of scarlet fever was 21% higher in males than females, adjusting for age group and year (aIRR, 1.21, 95%CI 1.09–1.34) (Table 1). Among males and females, there was no significant difference in the proportion of scarlet fever cases by age (Table 2, Supplementary Results). The proportion of males and females admitted to hospital, managed in a metropolitan or rural location, and their length of hospital stay were also similar. Annual incidence of scarlet fever was highest in those aged <5 years and 5–9 years (19.3 and 14.8 cases per 100 000, respectively) (Table 1).

## Discussion

To our knowledge, this is the first longitudinal population-based study of scarlet fever in Australia. We found ED presentations and hospitalizations for scarlet fever in Victoria were stable from 2007 to 2017, ranging from 1.9 to 3.1 cases per 100 000. Whilst annual incidence rates did not increase, incidence in Victoria was similar to China, which experienced a scarlet fever outbreak during this time. The incidence of scarlet fever in China was 4.14 per 100 000 from 2011 to 2016, which was 2.6 times higher than during 2003–2010 prior to the outbreak when the incidence was 1.58 per 100 000 [2]. In contrast, scarlet fever notification rates in England and Wales were higher during the scarlet fever resurgence from 2013 to 2016, increasing from 8 to 33 per 100 000 [1].

Whilst our findings demonstrate incidence of ED presentations and hospitalizations for scarlet fever were stable in Victoria over a > 10-year period, additional information would be useful. Future studies could analyze scarlet fever presentations within Australian primary care registries, given most cases present to general practice alongside the ED [9]. This could give a more accurate estimate of disease incidence in Victoria, as our analysis included only cases who presented to ED or were hospitalized. Furthermore, this study did not include microbiological analysis of scarlet fever cases. We were unable to retrieve data on the GAS serotype for scarlet fever cases (e.g., from throat swabs taken and analyzed) to investigate a potential association between particular GAS strains and scarlet fever.

In China, *emm*12 has been a common *emm*-type causing scarlet fever [2]. The finding of specific exotoxin prophage genes (such as *ssa* and *speC*) and antibiotic-resistant elements (such as macrolides and tetracyclines) amongst *emm*12 isolates could explain the potential selection and expansion of this lineage [2]. Alongside *emm*12, in the United Kingdom, other *emm*-types frequently associated with scarlet fever are *emm*1, *emm*3 and *emm*4 [1]. A new *emm*1 clonal lineage – M1_UK_ – has been particularly linked with seasonal surges of scarlet fever and outbreaks of invasive GAS disease in the United Kingdom [10].

Interestingly, this same M1_UK_ variant has recently become established in Australia [11], which may suggest Australia is at risk of a scarlet fever epidemic, as experienced in England, Wales, China and Hong Kong [1–3], where scarlet fever is notifiable. Thus, clinicians and public health officials should remain vigilant for scarlet fever. Despite the clear epidemic risk, scarlet fever is not notifiable in Australia. To aid early detection and management of potential future outbreaks in Australia, scarlet fever could be made notifiable. However, since scarlet fever is more often a clinical, rather than laboratory diagnosis, alternatives such as adding scarlet fever to general practice-based or ED sentinel surveillance networks could also be considered.

## Supporting information

Phakey et al. supplementary material Phakey et al. supplementary material

## Data Availability

The datasets generated during and/or analyzed during the current study are available from the corresponding author upon reasonable request.

## Supplementary material

For supplementary material accompanying this paper visit http://doi.org/10.1017/S0950268824001298. click here to view supplementary material
