# Supplementary material for: Epidemiology of scarlet fever in Victoria, Australia, 2007–2017
Source: Epidemiol Infect. 2024 Oct 4;152:e116. doi: 10.1017/S0950268824001298 (PMC11450502; doi:10.1017/S0950268824001298)
Supplement: Phakey et al. supplementary material [file S0950268824001298sup001.docx]

# *Epidemiology and Infection* – Epidemiology of scarlet fever in Victoria, Australia, 2007–2017. Sachin Phakey, Patricia T. Campbell, Katherine B. Gibney.

# Supplementary Material

**Supplementary Methods:**

The Victorian Emergency Minimum Dataset (VEMD) contains data on presentations to Victorian public hospitals with a designated emergency department (ED), including month and year of presentation, diagnoses for presentation, hospital location, as well as the individual’s age group, sex and preferred language (1). The Victorian Admitted Episodes Dataset (VAED) contains similar data on all hospitalisations to Victorian public and private hospitals (2). The National Cause of Death Unit Record File (CODURF) contains data relating to deaths recorded by the Victorian Registry of Births, Deaths and Marriages (3).

The Centre for Victorian Data Linkage extracted de-identified line-listed data from the VEMD, VAED and CODURF using International Statistical Classification of Disease and Related Health Problems, Tenth Revision (ICD-10) system codes relating to Group A *Streptococcus*. Records extracted were linked by assigning participants appearing more than once in any of the three datasets an encrypted numerical identifier.

Scarlet fever cases were identified from the three datasets using its ICD-10 (‘A38’) code. To avoid overestimating incident cases, cases in individuals with repeat ED presentations and hospital admissions within the same or following month were deemed to be related to the same episode of scarlet fever, and were only counted once. The VEMD, VAED and CODURF datasets were merged to identify both non-hospitalised (VEMD only) and hospitalised (VAED) scarlet fever cases, and deaths with scarlet fever (Supplementary Methods, Figure). There were 142 cases with an ICD-10 A38 diagnostic code for scarlet fever in both the VEMD and VAED, which were classified as hospitalised scarlet fever cases.

When calculating annual incidence rates of scarlet fever, Australian Bureau of Statistics mid-year estimated resident population data was used as the denominator (4). Seasonal variation was investigated by examining scarlet fever cases by year and calendar month (winter, June–August; spring, September–November; summer, December–February; and autumn, March–May), and formally tested using Edwards’ test, a goodness-of-fit test where an arbitrary seasonal curve with presumed fluctuations is fitted to a variable population at risk of disease (5).

Data analyses were conducted using Stata IC 15.1 Data Analysis and Statistical Software (StataCorp LP, College Station, TX, USA).


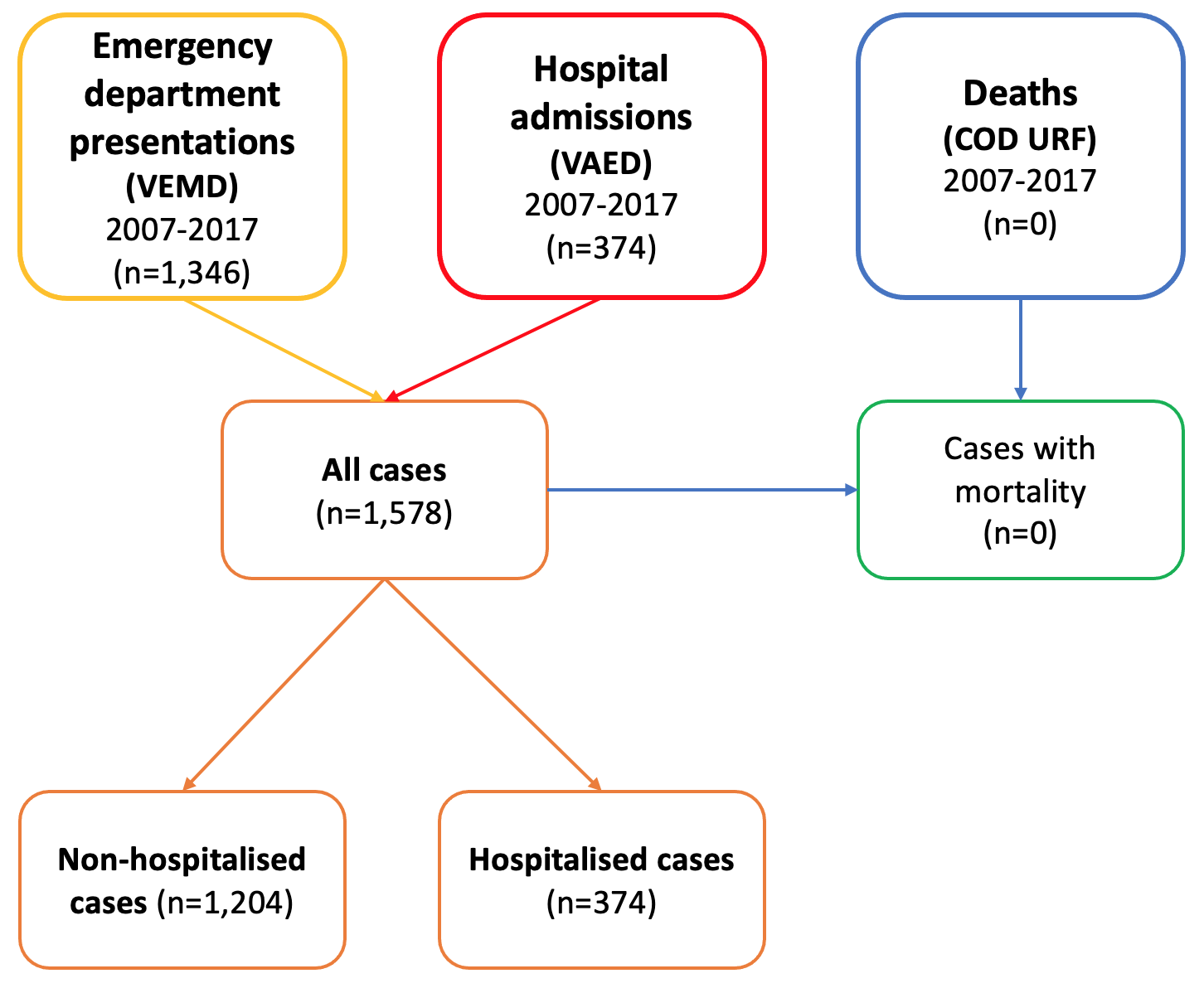


**Figure: Data merging process for scarlet fever emergency department presentations (Victorian Emergency Minimum Dataset, VEMD), hospitalisations (Victorian Admitted Episodes Dataset, VAED) and deaths (Cause of Death Unit Record File, CODURF)**

References:

1. Victorian Government Department of Health and Human Services. VEMD user manual 2021-22 sections 1 to 6. Victoria: Victorian Government; 2021. Updated 15 June 2021. <https://www.health.vic.gov.au/publications/vemd-user-manual-2021-22-sections-1-to-6> (viewed December 2021)

2. Victorian Government Department of Health and Human Services. VAED manual 2021-22 (all sections). Victoria: Victorian Government; 2021. Updated 8 June 2021. <https://www.health.vic.gov.au/publications/vaed-manual-2021-22-all-sections> (viewed December 2021)

3. Australian Institute of Health and Welfare. Mortality over regions and time (MORT) books. Canberra: Australian Government; 2021. Updated 25 June 2021. <https://www.aihw.gov.au/reports/life-expectancy-death/mort-books/contents/explanatory-notes> (viewed October 2021)

4. Australian Bureau of Statistics. 3101.0 National, state and territory population. Table 4. Estimated resident population, states and territories (number). Canberra: ABS; 2021. Updated 15 December 2021. <https://www.abs.gov.au/statistics/people/population> (viewed December 2021)

5. Walter SD, Elwood JM. (1975) A test for seasonality of events with a variable population at risk. *Br J Prev Soc Med*.;29:18-21.

**Supplementary Results:**

| **Supplementary Table 1: Characteristics of scarlet fever emergency department presentations and hospitalisations in Victoria, Australia, 2007–2017** | | | | | | |
| --- | --- | --- | --- | --- | --- | --- |
|  | **Total cases**  **(n=1,578)** | | **Hospitalised cases (n=374)** | | **Emergency department cases (n=1,204)** | |
|  | **n (%)** | | **n (%)** | | **n (%)** | |
| **Age group** (years) |  | |  | |  | |
| 0–4 | 775 | (49.1) | 172 | (46.0) | 603 | (50.1) |
| 5–9 | 569 | (36.1) | 142 | (38.0) | 427 | (35.5) |
| 10–14 | 72 | (4.6) | 18 | (4.8) | 54 | (4.5) |
| 15–19 | 67 | (4.3) | 16 | (4.3) | 51 | (4.2) |
| 20–24 | 29 | (1.8) | 10 | (2.7) | 19 | (1.6) |
| 25–29 | 11 | (0.7) | 3 | (0.8) | 8 | (0.7) |
| 30–34 | 18 | (1.1) | 5 | (1.3) | 13 | (1.1) |
| 35–39 | 11 | (0.7) | 2 | (0.5) | 9 | (0.8) |
| 40–54 | 11 | (0.7) | 4 | (1.1) | 7 | (0.6) |
| 55–94 | 15 | (0.9) | 2 | (0.5) | 13 | (1.1) |
|  |  |  |  |  |  |  |
| **Sex** |  |  |  |  |  |  |
| Male | 882 | (55.9) | 209 | (55.9) | 673 | (55.9) |
| Female | 696 | (44.1) | 165 | (44.1) | 531 | (44.1) |
|  |  |  |  |  |  |  |
| **Hospital location^a^** |  |  |  |  |  |  |
| Metropolitan | 1,091 | (69.1) | 268 | (71.7) | 823 | (73.5) |
| Rural | 402 | (25.5) | 106 | (28.3) | 296 | (26.5) |
|  |  |  |  |  |  |  |
| **Length of hospital stay** (days) |  |  |  |  |  |  |
| 0 (no hospital stay) | 1,204 | (76.3) |  |  | 1,204 | (100) |
| 1 (without overnight stay) | 80 | (5.1) | 80 | (21.4) |  |  |
| 1 (with overnight stay) | 117 | (7.4) | 117 | (31.3) |  |  |
| 2 | 91 | (5.8) | 91 | (24.3) |  |  |
| 3 | 57 | (3.6) | 57 | (15.2) |  |  |
| ≥4 | 29 | (1.8) | 29 | (7.8) |  |  |
|  |  |  |  |  |  |  |
| ^a^There were 85 emergency department presentations with scarlet fever whose hospital location (metropolitan vs rural) was not able to be determined | | | | | | |

| **Supplementary Table 2: Characteristics of fever emergency department presentations and hospitalisations in Victoria, Australia, 2007–2017 (n=1,578), by sex** | | | | |
| --- | --- | --- | --- | --- |
|  | **Male**  **(n=882)** | | **Female**  **(n=696)** | |
|  | **n (%)** | | **n (%)** | |
| **Age group** (years) |  | |  | |
| 0–4 | 449 | (50.9) | 326 | (46.8) |
| 5–9 | 310 | (35.1) | 259 | (37.2) |
| 10–14 | 49 | (5.6) | 23 | (3.3) |
| 15–19 | 36 | (4.1) | 31 | (4.5) |
| 20–34 | 24 | (2.7) | 34 | (4.9) |
| ≥35–39 | 14 | (1.6) | 23 | (3.3) |
|  |  |  |  |  |
| **Hospital admission** |  |  |  |  |
| Hospitalised | 209 | (23.7) | 165 | (23.7) |
| Emergency department | 673 | (76.3) | 531 | (76.3) |
|  |  |  |  |  |
| **Hospital location^a^** |  |  |  |  |
| Metropolitan | 615 | (69.7) | 476 | (68.4) |
| Rural | 224 | (25.4) | 178 | (25.6) |
|  |  |  |  |  |
| **Length of hospital stay** (days) |  |  |  |  |
| 0 (no hospital stay) | 673 | (76.3) | 531 | (76.3) |
| 1 (without overnight stay) | 36 | (4.1) | 44 | (6.3) |
| 1 (with overnight stay) | 71 | (8.0) | 46 | (6.6) |
| 2 | 50 | (5.7) | 41 | (5.9) |
| 3 | 32 | (3.6) | 25 | (3.6) |
| ≥4 | 20 | (2.3) | 9 | (1.3) |
|  |  |  |  |  |
| ^a^There were 85 emergency department presentations with scarlet fever whose hospital location (metropolitan vs rural) was not able to be determined | | | | |
